# A Fast and Sensitive Quantitative Lateral Flow Immunoassay for Cry1Ab Based on a Novel Signal Amplification Conjugate

**DOI:** 10.3390/s120911684

**Published:** 2012-08-27

**Authors:** Chunxiang Chen, Jian Wu

**Affiliations:** 1 Department of Control Science and Engineering, Zhejiang University, Hangzhou 310058, China; E-Mail: daocaoren198011@163.com; 2 Department of Biosytems Engineering and Food Science, Zhejiang University, Hangzhou 310058, China

**Keywords:** cry1Ab, fluorescence dye, lateral flow biosensor, polylysine

## Abstract

A novel lateral flow immunoassay (LFIA) signal amplification strategy for the detection of Cry1Ab based on amplification via a polylysine (PL) chain and biotin-streptavidin system (BSAS) is described. In this system, multiple fluorescence dyes (FL) were directly coated on the surface of PL and conjugated with antibody via the BSAS for construction of novel signal amplification (FLPL-BSAS-mAb1) conjugates, in which FL, PL and BSAS were employed to improve the sensitivity of LFIA. Compared with conventional LFIA, the sensitivity of FLPL-BSAS-mAb1-based LFIA was increased by approximately 100-fold. Quantified linearity was achieved in the value range of 0–1,000 pg/mL. The limit of detection (LOD) was reached 10 pg/mL after optimization of reaction conditions. To our knowledge, this represents one of the most sensitive LFIA for Cry1Ab yet reported. Furthermore, the detection time for this method was about 10 min. Therefore, it should be an attractive alternative compared to conventional immunoassays in routine control for Cry1Ab.

## 1. Introduction

Genetically modified organisms (GMOs) have been mainly developed for mass production of agricultural plants. The Cry toxins are insecticidal proteins, which are considered to be harmless and non-toxic to human being and animals. However, there are still safety concerns among consumers about the side effects GMOs might cause on ecosystems [1].

For the detection of Cry1Ab, the most commonly used formats are enzyme-linked immunosorbent assay (ELISA) [1–4] and lateral flow immunoassay (LFIA) [5], while various innovative analytical techniques have also been developed for quantitative or qualitative detection of Cry1Ab protein [6–14]. However, the main drawback of ELISA is the relatively long assay time required, large-scale instruments and professional operating techniques. Conventional LFIA often suffers from poor quantitative discrimination and low analytical sensitivity. Therefore, it is of crucial importance to establish a rapid testing methodology for monitoring Cry toxins.

In the past decades, several methods with different materials used as labels have been tested to increase the sensitivity for immunoassay, including fluorescence dye [15–17], liposomes [18–22], quantum dots (QDs) [23–27], polymers (dextran and polylysine chains) [28–31] and particles such as enzyme-gold nanoparticles [32], silica nanoparticles [33–38], superparamagnetic nanoparticles [39–41], polystyrene microparticles [42,43] and fluorescent europium(III) nanoparticles [44]. To overcome the limitations of traditional LFIA, the nanoparticle-based LFIA for signal amplification have achieved notable progress and improved sensing performance in a variety of biosensor systems. However, the sensitivity of LFIA cannot meet all demands from a variety of detection problems in food and environment nowadays. Thus, new kinds of signal amplification systems need to be explored.

Here, we present a novel signal amplification strategy in LFIA, which adopts three amplification steps: (a) biotin-streptavidin amplification; (b) polylysine amplification; (c) fluorescence dye signal amplification. The biotin-streptavidin system (BSAS) has been widely applied in immunohistochemistry and immunoassay for its high specificity and strong affinity [45,46]. Streptavidin (SA) contains four binding sites with an extraordinarily high affinity for biotin.

In this paper, we explored the use of this novel signal amplification conjugate as label for direct electronic signal measurement in LFIA. This efficient way to increase the sensitivity was achieved by amplification of the signals, which were generated from the fluorescence dye-antibody conjugate with a high fluorescence dye-to-antibody ratio. When FLPL-BSAS-mAb1 conjugate is bound to one antigen, tens or hundreds of fluorescence dye molecules would bind to a single antigen, consequently leading to signal amplification. In this assay, the resulting conjugates achieved a detection limit 100-fold lower than that of the magnetic beads-based ELISA [13] and gold-based LFIA [5]. The influence of some important parameters such as the type of nitrocellulose (NC) membrane, the structure of FLPL-BSAS-mAb1 conjugates and detection time of the present method were investigated in detail. Furthermore, the analytical performance of FLPL-BSAS-mAb1-based LFIA was further evaluated and its precision was also discussed.

## 2. Materials and Methods

### 2.1. Reagents and Materials

A nitrocellulose (NC) membrane, absorbent pad, sample pad, conjugate pad, and backing cards were purchased from Millipore (Bendford, MA, USA). Purified Cry1Ab protein, rabbit polyclonal antibody against Cry1Ab (pAb2), mouse monoclonal antibody against Cry1Ab (mAb1) and Bt Cry1Ab/1Ac/1F ELISA Kit were obtained from Abraxis LLC (Warminster, PA, USA), while Atto 647N (λ_absmax_ = 644 nm, λ_emmax_ = 669 nm), polylysine (30–70 KD), bovine serum albumin (BSA), N-(3-dimethylaminopropyl)-N′-ethylcarbodiimide hydrochloride (EDC), N-hydroxysuccinimide (NHS), streptavidin (SA), biotin and dimethyl sulfoxide (DMSO) were from Sigma (St. Louis, MO, USA). Other Cry proteins (Cry1C, Cry2A, Cry3A) were from Agdia Inc. (ElKart, IN, USA). Goat anti-rabbit IgG (GAR, >95%), rabbit IgG (RIgG, >95%) were obtained from Longji (Hangzhou, China). Dialysis tubing (20 KD) was from Spectrum Labs (Rancho Dominguez, CA, USA). All other analytical purified reagents were purchased domestically without further treatment or purification.

### 2.2. Apparatus

An XYZ Biostrip Dispenser and CM 4000 Cutter were purchased from Bio-Dot (Irvine, CA, USA). A portable fluorescence strip reader ESE-Quant FLUO was purchased from Invitrogen (Carlsbad, CA, USA). The ultracentrifuge is from Heraeus Biofuge Stratos (Sollentum, Germany). The SepectraMax M5 multi-mode microplate reader was from Molecular Devices (Sunnyvale, CA, USA).

### 2.3. Preparation of FLPL-BSAS-mAb1 Conjugates

#### 2.3.1. Preparation FLPL and RIgG-FL Conjugates

Briefly, biotin (2.44 mg) together with EDC (19.14 mg) and NHS (11.49 mg) were dissolved in 0.01 M phosphate buffer saline (PBS, pH 7.4, 0.1 mL) and stirred for 15 min at room temperature (RT). This solution was then added dropwise to polylysine (PL) solution (polylysine: 450 mg in 1 mL of 0.01 M PBS at pH 7.2) so the PL: biotin molar ratio was 1:1. The mixture was stirred at RT for 1 h and excessive unreacted biotin and ions in aqueous solution were removed by size exclusion (5 KD) column chromatography against PBS buffer (0.01 M, pH 7.4). Then the biotin-labeled PL was labeled with Atto 647N according to the Atto 647N protein labeling kit protocol from Sigma. Firstly, the solution of biotin-labeled PL was added with sodium bicarbonate buffer solution (pH 9.5), adjusted to pH 8.5–9.0 and transferred to Atto 647N (1 mg Atto 647N dissolved in 10 μL DMSO), incubated at room temperature under gentle shaking for 2 h, followed by being separated conjugates from free dye by a size-exclusion column. Finally, the Atto 647N labeled polylysine (FLPL conjugate) fraction was collected and stored at 4 °C. The rabbit polyclonal antibody-Atto 647N conjugate (RIgG-FL) was obtained and purified with the same process.

#### 2.3.2. Preparation of Biotinylated-mAb1

Biotinylation of Mouse monoclonal antibody (mAb1) was performed as described in Section 2.3.1. The molar ratio of biotin to mAb1 was 2:1. After labeling, excessive unreacted biotin and ions in aqueous solution were removed by dialysis against PBS buffer (0.01 M, pH 7.4) for 2 days. Then, it was stored in −20 °C until use.

#### 2.3.3. Preparation of FLPL-BSAS-mAb1 Conjugate

**Figure 1.**
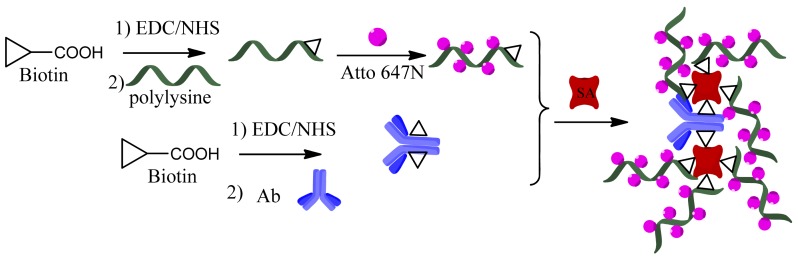
Preparation process of FLPL-BSAS-mAb1 conjugate.

The biotinylated-mAb1, streptavidin and FLPL conjugate were mixed in a molar ratio equal to 1:1:3 or 1:2:6 (Table 1). The conjugating mechanism of FLPL-BSAS-mAb1 conjugates was shown in Figure 1. Finally, FLPL-BSAS-mAb1 conjugate and RIgG-FL (1:1, v/v) were dispersed in stock solution (1% BSA and 0.005% sodium azide in 0.01 M PBS pH 7.4), respectively, and kept at 4 °C until use.

### 2.4. Preparation of Lateral Flow Immunoassay System

**Figure 2.**
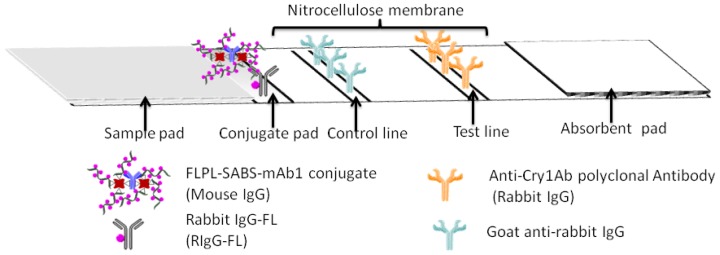
Schematic diagrams of FLPL-BSAS-mAb1 based LFIA.

The main body of the test strip consisted of five parts, including plastic backing, sample pad, conjugate pad, absorbent pad and NC membrane (Figure 2). Every component of the strip should be given a pretreatment described as follows: the NC membrane was attached to a plastic backing layer for cutting and handling. The pAb2 and GAR were immobilized at test line (T line) and control line (C line), respectively. The glass fiber was cut into two sizes 0.5 cm × 0.4 cm and 2.2 cm × 0.4 cm for the conjugate pad and sample pad. Conjugate pad contained FLPL-BSAS-mAb1 conjugate and RIgG-FL diluted by 0.01 M PBS buffer (pH 7.4) containing BSA (0.5%, w/v) and sucrose (3%, w/v). Sample pad was pretreated with BSA (3%, w/v) and Tween-20 (0.5%, w/v). Absorbent pad was 2.2 cm × 0.4 cm in size, attached to the top side of the strip.

### 2.5. Lateral Flow Test Procedure

Prior to the immunoassay, varying concentration of Cry1Ab standard solutions ranging from 0 to 1,000 pg/mL, were prepared by dilution in PBS buffer. One hundred μL of sample was loaded onto the sample pad of the strip, the test cartridge was inserted into the portable fluorescence strip reader after 10 min. The intensities of fluorescence conjugates on the detection zone were scanned and converted to area values. All experiments were performed in triplicate, and the average of the triplicates was used for the analysis.

### 2.6. Optimization Sensitivity of LFIA

#### 2.6.1. Optimization of FLPL-BSAS-mAb1 Conjugate

As described in Section 2.3.3, the biotinylated-mAb1, streptavidin and FLPL conjugate were mixed in different ratios and two different mixing orders were tested (see Table 1). In order to obtained higher signal amplification conjugate, different conjugate procedures were used in preparation.

#### 2.6.2. Optimization of NC Membrane

Five kinds of NC membranes (Millipore HF090, Millipore HF135, Sartourius CN95, Sartourius CN 140 and Whatman AE99) from different manufacturers were tested, respectively, according to the procedure described in Section 2.5. In this investigation, C line was coated with 1 mg/mL GAR, and T line was coated with 1 mg/mL pAb2. PBS buffer (10 mM, pH 7.4) was used as negative sample. After chromatography for 10 min, signals at the T and C line were measured.

#### 2.6.3. Optimization of Detection Time

Standard solution (1,000 pg/mL) was applied on the sample pad and allowed to migrate across the NC membrane. Signals of T line and C line were read every minute for 12 min.

### 2.7. Sample Preparation for Immunoassay Methods

Non-GM maize was purchased from a local market in Hangzhou, China, and verified with Bt Cry1Ab/1Ac/1F ELISA Kit. One hundred mg of the obtained non-GM maize flour was mixed with 1 mL of the extraction buffer (Tris-borate buffer, pH 7.5) and spiked with known amounts of Cry1Ab to obtain samples with different concentrations of 0, 5, 10, 30, 60, 125, 250, 500, 1,000 pg/mL [4]. Sample without Cry1Ab was used as a blank. Each mixture was shaken for 30 min and centrifuged at 8,000 g for 30 min to remove insoluble material. The liquid protein extract was used in the assay.

## 3. Results and Discussion

### 3.1. Principle of FLPL-BSAS-mAb1 Based LFIA

FLPL-BSAS-mAb1-based LFIA combined the unique optical properties of FL with a signal amplification system for ultrasensitive detection of Cry1Ab. The protocol of FLPL-BSAS-mAb1 conjugate preparation was illustrated in Figure 1 and set-up of the LFIA in Figure 2. Standard solution containing target Cry1Ab was added on the sample pad, migrated by capillary action and passed the NC membrane. Finally, the Cry1Ab were captured by the pAb2 on the T line, while FLPL-BSAS-mAb1 conjugate were captured by Cry1Ab. The FL intensity at the T line was proportional to the concentration of Cry1Ab protein and scanned by a portable fluorescence strip reader. The GAR was fixed at control line served as an internal control after the reaction with RIgG-FL.

**Figure 3.**
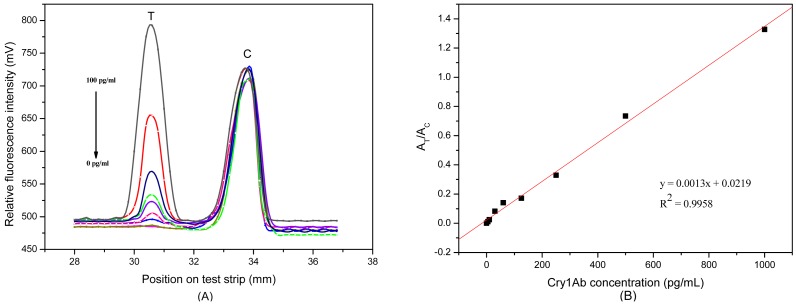
(**A**) Response for Cry1Ab concentration of 0, 5, 10, 30, 60, 125, 250, 500, and 1,000 pg/mL in buffer; (**B**) The calibration curve obtained from the area ration (A_T_/A_C_) against the concentration of Cry1Ab in buffer.

Figure 3(A) shows the relative fluorescence intensity (RFI) profile from the scanned test strip. The first peak and the second peak displayed RFI on T and C line, respectively, as plotted in the y-axis. The distance of strip was plotted in x-axis. The RFI of the C line was almost unchanged in different samples, indicating that the C line is functioning as a good internal standard.

### 3.2. Optimization Sensitivity of LFIA

#### 3.2.1. Optimization of FLPL-BSAS-mAb1 Conjugate

The use of polylysine with a big amount of primary amino was vital for signal amplification in LFIA, which in turn would influence the loading amount of Atto 647N on the conjugate. The biotinylated-mAb1 and FLPL conjugate self-assembled via the streptavidin–biotin system. We compared the analytical performances of four types of conjugates prepared through different conjugation procedures (Table 1). In the presence of Cry1Ab (1,000 pg/mL), there was a very weak response observed with Type 1 conjugate (T1) and the highest responses were achieved by Type 3 and Type 4 conjugates (T3 and T4).

**Figure 4.**
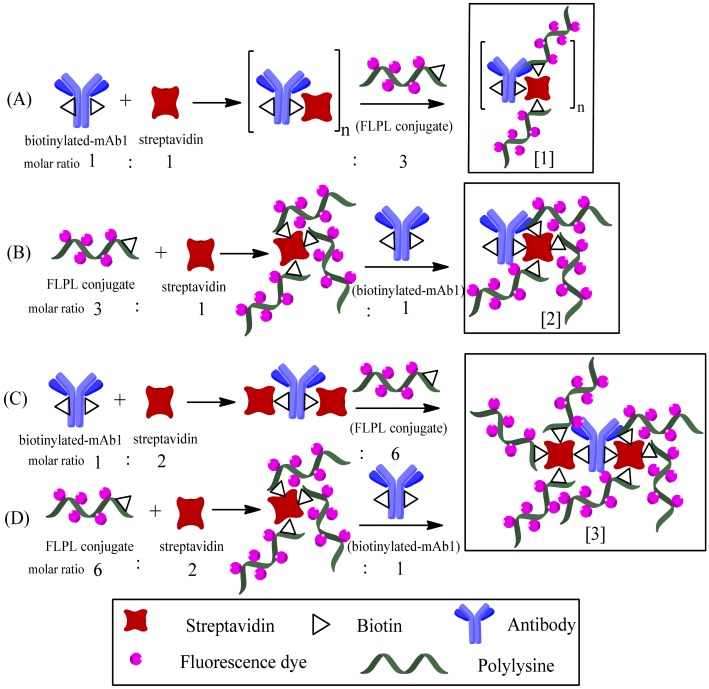
Chemical structures of various FLPL-BSAS-mAb1 conjugates.

Figure 4 illustrates the sequential steps and structures of various conjugates prepared by chemical modification of mAb1. T3 and T4 had similar signal intensity and 2-fold higher than type 2 conjugate (T2), indicating they may have similar structures. In addition, T3 and T4 demonstrated a 60-fold higher intensity compared to T1. Therefore, we propose three kinds of structures for these conjugates. In these tests, the signal response of T1 accumulated on the bottom of the NC membrane, that may be caused by the steric hindrance effect of a polymer, as only a few tiny polymers could migrate across the NC membrane (Figure 4, panel A), so T1 might be structure 1. T2 might be structure 2, while T3 and T4 might be structure 3, because structure 3 potentially has 2-fold more FL molecules conjugated on mAb1 and would be expected to generate a 2-fold higher response compared to structure 2 (Figure 4, panels B, C and D, respectively). Thus, structure 3 was used to fix on conjugate pad for the following experiments. Furthermore, we have attempted to apply longer polylysine (70−150 KD) to construct higher bright conjugates, but the results indicated that conjugates with longer polylysine chains cannot for pass through the NC membrane, because they would clog in the membrane pores (data not shown).

#### 3.2.2. Optimization of NC Membrane

**Figure 5.**
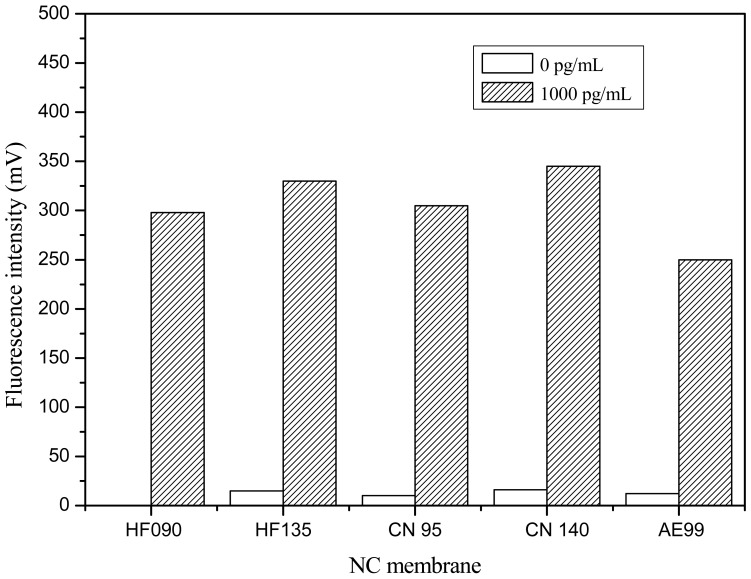
Responses at T lines on five different NC membranes (CN 95, HF 090, CN 140, HF 135, AE 99).

Responses at the T lines on five NC membranes were compared in Figure 5. Responses of strips constructed with Sartorius CN 140 membrane were the highest, while the response of strips constructed from Whatman AE 99 membrane were the lowest in the presence of 1,000 pg/mL Cry1Ab. In detection of 0 ppt Cry1Ab, a little response could be observed on Millipore HF135, Sartourius CN95, Sartourius CN 140 and WhatmanAE99, because a few of the FLPL-BSAS-mAb1 conjugates would physically clog the small size of pores of these NC membranes, but almost no response could be observed on the Millipore HF 090 membrane in detection of 0 ppt Cry1Ab. With consideration both of the responses on 0 and 1,000 pg/mL, Millipore HF 090 was selected as the most suitable NC membrane for strip preparation in the following experiments.

#### 3.2.3. Optimization of Detection Time

**Figure 6.**
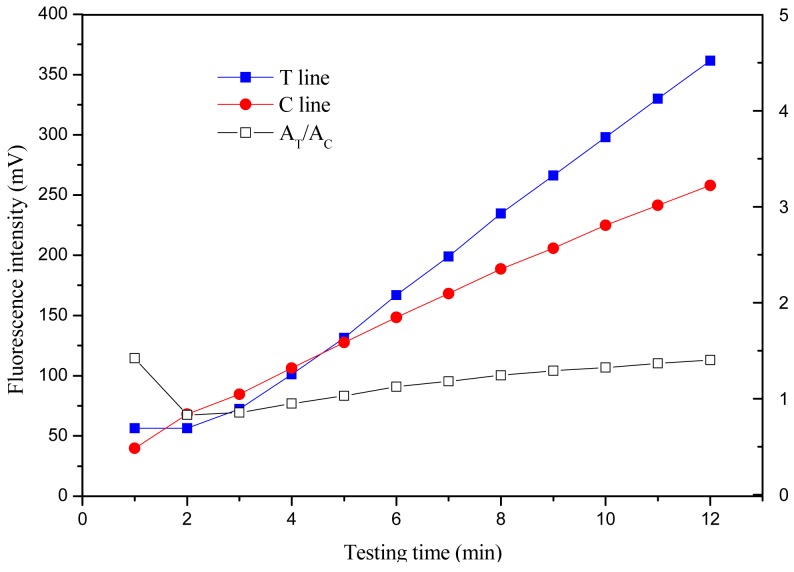
Trend of signals on T line and C line with time at 1,000 pg/mL.

The time-intensity curves were obtained by measuring the signals of the T and C lines every minute for 12 min after standard solution addition. The time-intensity curves and time-signal ratio A_T_/A_C_ curve at one concentration of 1,000 pg/mL are shown in Figure 6, respectively.

The T line and C line signals increased rapidly during the first 25 min, between 25 and 45 min, the signals increased slowly and entered a plateau (data not shown). The time-signal ratio A_T_/A_C_ curves of the first 12 min have been observed, and indicated that A_T_/A_C_ would vary in the first 9 min, then gradually plateau, indicating the signal intensity was relatively stable for analysis (reading) over 9−12 min. With consideration of signal intensity for low concentration sample and the shortest time for detection, the detection time was considered as 10 min.

### 3.3. Method Evaluation

#### 3.3.1. Sensitivity

After optimizing the signal amplification system, a series of Cry1Ab samples were tested by the FLPL-BSAS-mAb1-based LFIA to obtain a standard curve. Quantitative detection data is illustrated in Figure 3(A). There was an obvious increase in signals at the T line with the increasing Cry1Ab concentration. A standard curve for Cry1Ab was plotted by the signal ratio A_T_/A_C_ at different Cry1Ab concentrations (Figure 3(B)). It showed good linearity in the range between 0 and 1,000 pg/mL; the equation was:

(1) [y=0.0013x+0.0219]

while the correlation coeffcients were calculated to be 0.9958. The coefficients of variation (CV) values were both lower than 10%. The cut off level for all detection systems was calculated by the following equation:

(2) [3∗SD background+average background]

A detection limit of 10 pg/mL was achieved, which was 100 times lower than that of the magnetic beads- based ELISA [13] and gold-based LFIA [5]. This suggested that the proposed method is highly sensitive, especially for detection of biomolecules at low levels.

#### 3.3.2. Specificity

The cross-reactivity (CR) and specificity of FLPL-BSAS-mAb1 based LFIA for Cry1Ab was assessed with available different samples: Cry1C, Cry2A, Cry3A. It was checked with different dilutions of above proteins but there was no cross-reactivity with those three transgenic proteins, indicating that this assay can be used for unique quantification of Cry1Ab protein.

#### 3.3.3. Accuracy Evaluation

As ingredients in food are more complicated than the PBS buffer, Cry1Ab recovery rates in flour matrix were calculated to evaluate the influence of matrix on detection of target cry1Ab. It was calculated using the formula:

(3) [(observed concentration/spiked concentration)×100]

and expressed in percent. As shown in Table 2, the recoveries were ranged from 80.05% to 109.69% with CVs of 3.8–9.5% in signal-amplification-based LFIA.

## 4. Conclusions

In conclusion, in this paper, one signal-amplification-based LFIA for ultrasensitive detection of Cry1Ab has been developed with very high analytical sensitivity (10 pg/mL) and short turnaround time (10 min). The signal of FLPL-BSAS-mAb1 conjugate was enlarged by increasing the amount of fluorescence dye loading on antibody via a polylysine chain and biotin–streptavidin system. It was found that PL and BSAS played very important roles to improve the sensitivity of the assay. Furthermore, optimization of factors which may affect the detection sensitivity and method assessment were also described. This method was applicable in a flour matrix. Therefore, FLPL-BSAS-mAb1-based LFIA has great potential to improve detection sensitivity, making is a useful technique for more sensitive and quantitative immunoassays.

## Figures and Tables

**Table 1.** Properties of FLPL-BSAS-mAb1 conjugate prepared with different conjugate types.

**Type No**	**Molar ratio**			**Mix order** ^a^^,^^b^	**Intensity** ^c^ **(mV)**

	**biotinylated-mAb1**	**streptavidin**	**FLPL conjugate**		
A	1	1	3	Method 1	10
**B**	1	1	3	Method 2	137
C	1	2	6	Method 1	298
D	1	2	6	Method 2	285

a Order 1: Method 1: streptavidin was added into the biotinylated-mAb1 solution prior to the addition of FLPL conjugate; Method 2: streptavidin was added into the FLPL conjugate solution prior to the addition of iotinylated-mAb1.

b Reaction time of each mix step: 30 min.

c The intensities of test line were obtained with a portable strip reader. Concentration of Cry1Ab: 1,000 pg/mL.

**Table 2.** Recovery and precision of FLPL-BSAS-mAb1 based LFIA spiked in flour sample (n = 5).

**Spiked Concentration (pg/mL)**	**Mean** ^a^ **(pg/mL)**	**SD**	**Recovery (%)**	**CV (%)**
50	40.02	1.52	80.05	3.8
200	210.18	13.03	105.09	6.2
500	480.95	36.06	96.19	7.5
800	877.52	83.36	109.69	9.5

a The mean and standard deviation values were calculated from five independent experiments.

## References

[1] Paula V., Steinkea K., Meyer H. (2008). Development and validation of a sensitive enzyme immunoassay for surveillance of Cry1Ab toxin in bovine blood plasma of cows fed Bt-maize (MON810). Anal. Chim. Acta..

[2] Wang S., Guo A.Y., Zheng W.J., Zhang Y., Qiao H., Kennedy I.R. (2007). Development of ELISA for the determination of transgenic Bt-Cottons using antibodies against Cry1Ac protein from Bacillus thuringiensis HD-73. Eng. Life. Sci..

[3] Nakajima O., Teshima R., Takagi K., Okunuki H., Sawada J. (2007). ELISA method for monitoring human serum IgE specific for Cry1Ab introduced into genetically modified corn. Regul. Toxicol. Pharm..

[4] Walschus U., Witt S., Wittmann C. (2002). Development of monoclonal antibodies against Cry1Ab protein from Bacillus thuringiensis and their application in an ELISA for detection of transgenic Bt-Maize. Food Agr. Immunol..

[5] Kumar R., Singh C.K., Kamle S., Sinha R.P., Bhatnagar R.K., Kachru D.N. (2010). Development of nanocolloidal gold based immunochromatographic assay forrapid detection of transgenic vegetative insecticidal protein in geneticallymodified crops. Food Chem..

[6] Volpe G., Ammid N.H., Moscone D., Occhigrossi L., Palleschi G. (2006). Development of an immunomagnetic electrochemical sensor for detection of BT-CRY1AB/CRY1AC proteins in genetically modified corn samples. Anal. Lett..

[7] Giovannoli C., Anfossi L., Baggiani C., Giraudi G. (2008). Binding properties of a monoclonal antibody against the Cry1Ab from Bacillus Thuringensis for the development of a capillary electrophoresis competitive immunoassay. Anal. Bioanal. Chem..

[8] Jang H.J., Cho I.H., Kim H.S., Jeon J.W., Hwang S.Y., Paek S.H. (2011). Development of a chemiluminometric immunosensor array for on-site monitoring of genetically modified organisms. Sens. Actuator B.

[9] Székács A., Lauber É., Takács E., Darvas B. (2010). Detection of Cry1Ab toxin in the leaves of MON 810 transgenic maize. Anal. Bioanal. Chem..

[10] Roda A., Mirasoli M., Guardigli M., Michelini E., Simoni P., Magliulo M. (2006). Development and validation of a sensitive and fast chemiluminescent enzyme immunoassay for the detectionof genetically modified maize. Anal. Bioanal. Chem..

[11] Ermolli M., Prospero A., Balla B., Querci M., Mazzeo A., Eede G.V.D. (2006). Development of an innovative immunoassay for CP4EPSPS and Cry1AB genetically modified protein detection and quantification. Food Addit. Contam..

[12] Fantozzi A., Ermolli M., Marini M., Scotti D., Balla B., Querci M., Langrell S.R.H., Eede G.V.D. (2007). First application of a microsphere-based immunoassay to the detection of genetically modified organisms (GMOs): Quantification of Cry1Ab protein in genetically modified maize. J. Agric. Food Chem..

[13] Li D.Y., Fan K., Wu J., Ying Y.B. (2011). Magnetic beads transfer based assay for CrylAb protein. Chin. J. Anal. Chem..

[14] Zhu X.L., Chen L.L., Shen P., Jia J.W., Zhang D.B., Yang L.T. (2011). High sensitive detection of Cry1Ab protein using a quantum dot-based fluorescence-linked immunosorbent assay. J. Agric. Food Chem..

[15] Pyo D.J. (2007). Comparison of fluorescence immunochromatographic assay strip and gold colloidal immunochromatographic assay strip for detection of microcystin. Anal. Lett..

[16] Oh S.W., Kim Y.M., Kim H.J., Kim S.J., Cho J.S., Choi E.Y. (2009). Point-of-care fluorescence immunoassay for prostate specific antigen. Clin. Chem. Acta.

[17] Yoo J.S., Jung Y.M., Hahn J.H., Pyo D.J. (2010). Quantitative analysis of a prostate-specific antigen in serum using fluorescence immunochromatography. J. Immunoassay Immunochem..

[18] Zaytseva N.V., Montagna R.A., Lee E.M., Baeumner A.J. (2004). Multi-analyte single-membrane biosensor for the serotype-specific detection of Dengue virus. Anal. Bioanal. Chem..

[19] Edwards K.A., Baeumner A.J. (2006). Liposomes in analyses. Talanta.

[20] Zhan W., Bard A.J. (2007). Electrogenerated chemiluminescence: Immunoassay of human C-reactive protein by using Ru(bpy)_3_^2+^-encapsulated liposomes as labels. Anal. Chem..

[21] Khreich N., Lamourette P., Lagoutte B., Ronco C., Franck X., Créminon C., Volland H. (2010). A fluorescent immunochromatographic test using immunoliposomes for detecting microcystins and nodularins. Anal. Bioanal. Chem..

[22] Khreich N., Lamourette P., Boutal H., Devilliers K., Créminon C., Volland H. (2008). Detection of Staphylococcus enterotoxin B using fluorescent immunoliposomes as label for immunochromatographic testing. Anal. Biochem..

[23] Zou Z.X., Du D., Wang J., Smith J.N., Timchalk C., Li Y.Q., Lin Y.H. (2010). Quantum dot-based immunochromatographic fluorescent biosensor for biomonitoring trichloropyridinol, a biomarker of exposure tochlorpyrifos. Anal. Chem..

[24] Chen Y.P., Ning B.A., Liu N., Feng Y., Liu Z., Liu X.Y., Gao Z.X. (2010). A rapid and sensitive fluoroimmunoassay based on quantum dot for the detection of chlorpyrifos residue in drinking water. J. Environ. Sci. Heal. B.

[25] Chen J.X., Xu F., Jiang H.Y., Hou Y.L., Rao Q.X., Guo P.J., Ding S.Y. (2009). A novel quantum dot-based fluoroimmunoassay method for detection of Enrofloxacin residue in chicken muscle tissue. Food Chem..

[26] Li Z.H., Wang Y., Wang J., Tang Z.W., Pounds J.G., Lin Y.H. (2010). Rapid and sensitive detection of protein biomarker using a portable fluorescence biosensor based on quantum dots and a lateral flow test strip. Anal. Chem..

[27] Yang H., Li D., He D., Guo Q., Wang K., Zhang X.Q., Huang P., Cui D.X. (2010). A noval quantum dots-based point of care test for syphilis. Nanoscale Res. Lett..

[28] Mateo C., Palomo J.M., Langen L.M.V., Rantwijk F.V., Sheldon R.A. (2004). A new, mild cross-linking methodology to prepare cross-linked enzyme aggregates. Biotechnol. Bioeng..

[29] Fuentes M., Segura R.L., Abian O., Betancor L., Hidalgo A., Mateo C., Fernandez-Lafuente R., Guisan J.M. (2004). Determination of protein-protein interactions through aldehyde-dextran intermolecular cross-linking. Proteomics.

[30] Dhawan S. (2002). Design construction of novel molecular conjugates for signal amplification (I): Conjugation of multiple horseradish peroxidase molecules to immunoglobulin via primary amines on lysine peptide chains. Peptides.

[31] Chang W.F., Wang S.J., Lai S.F., Shieh C.J., Hsiung K.P., Liu Y.C. (2011). Evaluation on the use of reactive dye-modified polylysine as the biomarker in immunochromatographic test application. Anal. Biochem..

[32] He Y.Q., Zhang S.Q., Zhang X.B., Baloda M., Gurung A.S., Xu H., Zhang X.J., Liu G.D. (2011). Ultrasensitive nucleic acid biosensor based on enzyme-gold nanoparticle dual label and lateral flow strip biosensor. Biosens. Bioelectron..

[33] Wu Y.F., Chen C.L., Liu S.Q. (2009). Enzyme-functionalized silica nanoparticles as sensitive labels in biosensing. Anal. Chem..

[34] Piao Y., Burns A., Kim J., Wiesner U., Hyeon T. (2008). Designed fabrication of silica-based nanostructured particle systems for nanomedicine applications. Adv. Funct. Mater..

[35] Chen L., Chen C., Li R., Li Y., Liu S. (2009). CdTe quantum dot functionalized silica nanosphere labels for ultrasensitive detection of biomarker. Chem. Commun..

[36] Wang J., Liu G., Engelhard M.H., Lin Y. (2006). Sensitive immunoassay of a biomarker tumor necrosis factor-α based on poly(guanine)-functionalized silica nanoparticle label. Anal. Chem..

[37] Ke R.Q., Yang W., Xia X.H., Xu Y., Li Q.G. (2010). Tandem conjugation of enzyme and antibody on silica nanoparticle for enzyme immunoassay. Anal. Biochem..

[38] Xia X.H., Xu Y., Zhao X.L., Li Q.G. (2009). Lateral flow immunoassay using europium chelate–loaded silica nanoparticles as labels. Chin. Chem..

[39] Wang Y.Y., Xu H., Wei M., Gu H.C., Xu Q.F., Zhu W. (2009). Study of superparamagnetic nanoparticles as labels in the quantitative lateral flow immunoassay. Mater. Sci. Eng..

[40] Zheng C., Wang X.C., Lu Y., Liu Y. (2012). Rapid detection of fish major allergen parvalbumin using superparamagnetic nanoparticle-based lateral flow immunoassay. Food Control..

[41] Tang D., Sauceda J.C., Lin Z., Ott S., Basova E., Goryacheva I., Biselli S., Lin J., Niessner R., Knopp D. (2009). Magnetic nanogold microspheres-based lateral-flow immunodipstick for rapid detection of aflatoxin B_2_ in food. Biosens. Bioelectron..

[42] Yan J.L., Estevez M.C., Smith J.E., Wang K.M., He X.X., Wang L., Tan W.H. (2007). Dye-doped nanoparticles for bioanalysis. Nano Today.

[43] Valanne A., Suojanen J., Peltonen J., Soukka T., Hanninen P., Harma H. (2009). Multiplesized europium(III) chelate-dyed polystyrene particles as donors in FRET: An application for sensitive protein quantification utilizing competitive adsorption. Analyst.

[44] Juntunen E., Myyryläinen T., Salminen T., Soukka T., Pettersson K. (2012). Performance of fluorescent europium (III) nanoparticles and colloidal gold reporters in lateral flow bioaffinity assay. Anal. Biochem..

[45] Liu R.P., Liu J.T., Xie L., Wang M.X., Luo J.P., Cai X.X. (2010). A fast and sensitive enzyme immunoassay for brain natriuretic peptide based on micro-magnetic probes strategy. Talanta.

[46] Sai N., Chen Y.P., Liu N., Yu G.G., Su P., Feng Y., Zhou Z.J., Liu X.Y., Zhou H.Y., Gao Z.X., Ning B.A. (2010). A sensitive immunoassay based on direct hapten coated format and biotin-streptavidin system for the detection of chloramphenicol. Talanta.

